# Vascular Strip Cholesteatoma-A Case Report 

**Published:** 2019-09

**Authors:** Ajay M Bhandarkar, Samarth Goyal, Manna Valiathan, Kailesh Pujary

**Affiliations:** 1 *Department of ENT, Kasturba Medical College, Manipal Academy of Higher Education, Madhavnagar Manipal, India.*; 2 *Undergraduate Student, Kasturba Medical College, Manipal Academy of Higher Education, Manipal, India.*; 3 *Department of Pathology, Kasturba Medical College, Manipal Academy of Higher Education, Manipal, India.*

**Keywords:** Cholesteatoma, Canal wall reconstruction, External Auditory Canal, Mastoidectomy

## Abstract

**Introduction:**

The incidence of cholesteatoma occurring as a result of tympanoplasty is extremely rare. Understanding the cause and preventing its occurrence in the future is the main intention of highlighting this peculiar presentation.

**Case Report:**

A 25-year-old woman presented with progressive hearing loss and blocked sensation in the left ear of one and a half months duration. Past history revealed a history of left myringoplasty six years prior to presentation. Clinical examination of the ear revealed a smooth, soft epithelium covered bulge in the lateral one-third of the floor and posterior wall of the left external auditory canal. HRCT and MRI of the temporal bone confirmed the presence of a soft tissue density in the mastoid. Pure tone audiometry revealed conductive hearing loss. She underwent mastoid exploration, removal of sac with soft wall reconstruction.

**Conclusion:**

Proper placement of the vascular strip with the skin lining the external auditory canal with approximation of the incision margins is essential to prevent iatrogenic cholesteatoma formation. Close follow-up is essential to prevent any recurrence and diffusion weighted MRI plays a vital role in detection of recurrence.

## Introduction

Cholesteatoma is a sac containing keratinising squamous epithelium presenting in the middle ear and mastoid with a propensity to erode bone and cause complications intracranially and extracranially (1,2). The incidence of cholesteatoma occurring at the site of vascular strip as a result of tympanoplasty is extremely rare and there is only one documented case series in literature which describes the development of vascular strip cholesteatoma (3). There is also one case series reported in literature which deals with cholesteatoma at the bony-cartilagenous junction following an intact canal wall mastoidectomy (1). Understanding the cause and preventing its occurrence in the future is the main intention of highlighting this peculiar presentation. We present a young lady with an atypical presentation of cholesteatoma in the external auditory canal which can be attributed to a past otological surgery.

## Case Report

**Fig 1:**
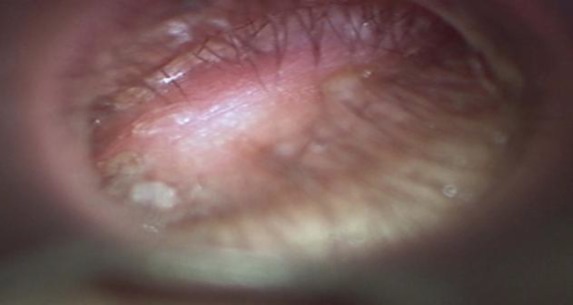
Epithelium covered bulge in the lateral part of external auditory canal

A 25-year-old woman presented to our out-patient department with progressive hearing loss and blocked sensation in the left ear for of one and a half months duration. There was no history of ear discharge, ear pain, vertigo and tinnitus. There was no history of nose or throat complaints. Past history revealed a history of left myringoplasty six years prior to presentation. Clinical examination of the ear revealed a smooth, soft epithelium covered bulge in the lateral one-third of the floor and posterior wall of the left external auditory canal which was sensitive to touch and did not bleed on touch (Fig.1). Examination of the nose, throat and neck was normal. Systemic examination was normal.

**Fig 2:**
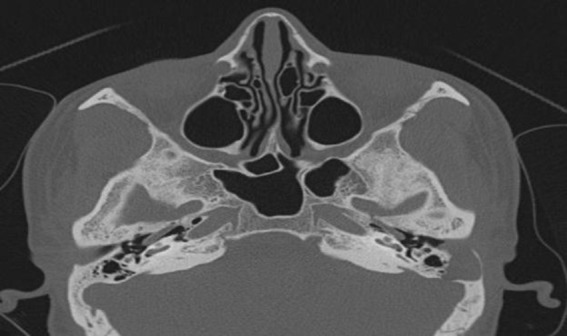
HRCT Temporal Bone revealing erosion of posterior wall of external auditory canal by Cholesteatoma

HRCT of the temporal bone revealed a soft tissue density in the lateral one-third of the external auditory canal eroding the posterior wall and extending into the mastoid bone impinging on the sigmoid sinus plate (Fig.2). 

**Fig 3a:**
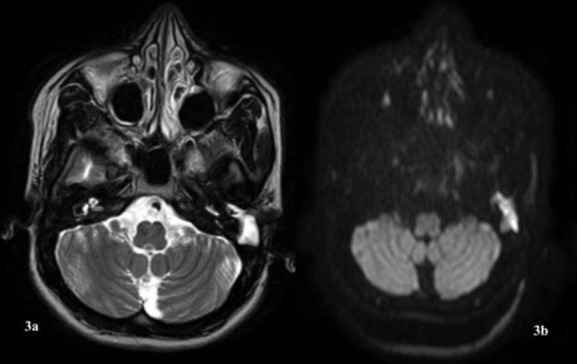
T2W Gadolinium MRI image showing a hyperintense density in the left external auditory canal and mastoid. **3b:** DWI MRI image showing a hyperintense density in the left external auditory canal.

MRI with Gadolinium contrast of the temporal bone revealed a well-defined lobulated T2W hyperintense lesion (Fig.3a) with partial suppression on FLAIR images which appeared mildly hyperintense on DWI images (Fig.3b), not showing post contrast enhancement in the left external auditory canal arising from the external auditory canal extending into the mastoid bone with scalloping with no obvious erosion in the left sigmoid sinus plate denoting the possibility of a benign cystic lesion. Pure tone audiometry revealed a moderate conductive hearing loss with an air-bone gap of 30 decibels.

**Fig 4:**
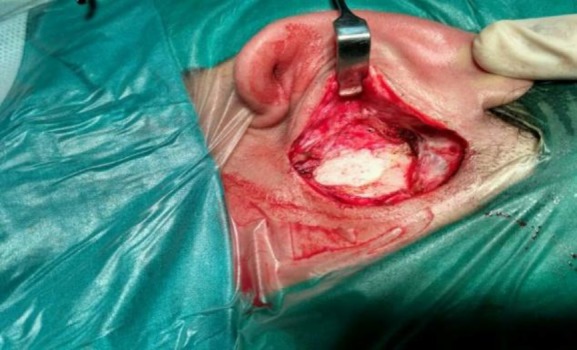
Intraoperative image showing the sac with cholesteatoma

The patient underwent left mastoid exploration under general anaesthesia. Postauricular incision with exposure of the mastoid cortex showed a well-defined sac with keratin debris in the mastoid which was originating from the bony cartilagenous region of the external auditory canal and eroding the mastoid cortex (Fig.4). 

The sigmoid sinus plate, mastoid antrum, dural plate and tympanic membrane were intact. The sac was removed in toto and sent for histopathological examination. The defect in the posterior external auditory canal was reconstructed with cartilage, temporalis fascia and periosteum.

**Fig 5:**
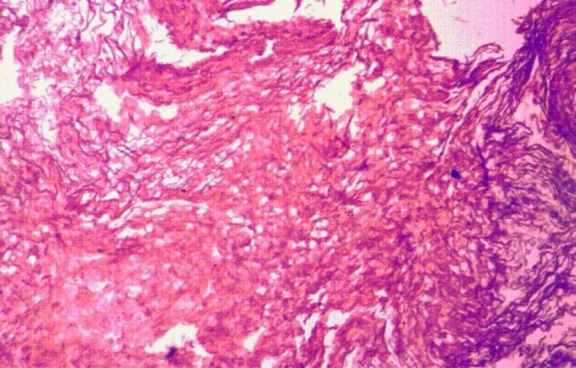
Histopathological image showing presence of keratin flakes and lamellated keratin and eroded fragments of bone along with fibro-collagenous stroma

Histopathological examination showed the presence of keratin flakes and lamellated keratin and eroded fragments of bone along with fibro-collagenous stroma. Keratinized stratified squamous epithelium overlying fibro-collagenous stroma was infiltrated with lymphoplasmacytic infiltrate, cystic macrophages and multinucleated giant cells consistent with features of cholesteatoma (Fig.5).

Follow-up after three months showed a well-delineated external auditory canal. Post-operative HRCT temporal bone at three months showed a well-healed cartilage graft surrounded by soft tissue without any evidence of residual disease.

## Discussion

Cholesteatoma has been described as a sac of epithelium containing desquamated keratin debris with a propensity to erode surrounding bone (1). Presence of such a sac in the external auditory canal has been termed as external auditory canal cholesteatoma(EACC). In 1850, Toynbee first described the clinical pathology but it was Scholefield in 1893 who termed it external auditory canal cholesteatoma. In 1980, Piepergerdes defined it as a clinical entity which precisely isolated it from other pathologies in the external auditory canal (2). 13 case series have been reported in literature on EACC. Only one case series stresses the fact of vascular strip cholesteatoma which is similar to our case report (3). The incidence of EACC is 0.19 to 0.3 per 100,000 population. However, the pediatric population has an incidence of 1.6 per 1000 population which manifest clinically (2-6).

EACC can be of 2 variants: primary and secondary. Primary EACC can be caused by first branchial cleft rudiment which creates a fistulous sinus and epithelium mass remnants in the fistulous sinus. It can also be caused by recurrent microtrauma or microangiopathy produced by cigarette smoking. Secondary EACC is either caused by obstruction or bony defects. Obstruction can be due to exostosis, nevi, stenosis or mycetomas. Bony defects can be post-inflammatory, post-traumatic, postoperative, radiogenic and post tumours. Sweeney et al suggested that vascular strip inversion during tympanoplasty surgery resulted in the formation of cholesteatoma. It can be a result of a shortened vascular strip, overthinning of the mastoid cortex and the influence of pressure negativity within the mastoid cavity (2,3,5). Cronin et al suggested that a shortened vascular strip caused chronic osteitis due to exposure of bone and entrapment of skin under the vascular strip leading to cholesteatoma (1). 

Otorrhea is the most common presentation followed by itching and fullness in the ear. On examination, the presence of keratin debris with a sac in the floor of the external auditory canal is the most common finding whereas, secondary EACC is commonly multifocal in nature. The presence of past history of surgery, repeated cotton-tipped applicator use, smoking and hearing aid use have to be determined to indicate the presence of predisposing factors for EACC. Our patient had a peculiar presentation of decreased hearing of short duration and examination revealed a smooth skin covered bulge in the posterior wall of the external auditory canal with the absence of keratin debris (1-4). However, our patient did have past history of surgery which raised the possibility of a cholesteatoma. Reduced hearing in our patient can be attributed to the occlusion by the smooth skin covered bulge which was occluding the external auditory canal.

HRCT of the temporal bone is useful in diagnosis. It detects the presence of soft tissue density with bone erosion (2,4). MRI with gadolinium contrast may be complementary if there is an intracranial extension of the sac. Diffusion-weighted MRI has proven to be the gold standard in recurrence or residual disease. Cholesteatomas are hyperintense on diffusion-weighted MRI, thereby proving a very sensitive utility in recurrence and recidivism (7).

Holt proposed a 3 stage classification based on the extension of the sac from the external auditory canal to the mastoid (8). Naim et al proposed a 4 stage classification based on the histopathology and local spread of the cholesteatoma (9). Early stages can be treated by conservative medical management including topical antibiotics and anti-inflammatory ointment. Stage 2 and above require surgical treatment in the form of sac excision and reconstruction. Surgical treatment can vary from a simple canalplasty to a canal wall down mastoidectomy with external auditory canal wall reconstruction with bone pate, cartilage or musculoperiosteal flap (1,2,6). In our case, we did an excision of the sac with external auditory canal wall reconstruction in 3 layers with temporalis fascia, cartilage and bone pate. 

Recurrence following surgical treatment for vascular strip cholesteatoma is extremely rare (1-3). There is no known complication of surgery for vascular strip cholesteatoma documented in literature till date. Prevention of vascular strip cholesteatoma is extremely important take-home message from this report. It can be ascertained by meticulous dissection of the vascular strip and elevation of the tympanomeatal flap. The vascular strip should be essentially approximated in close apposition to the tympanomeatal flap following tympanoplasty surgery. This will ensure proper healing without the accumulation of keratin debris thereby preventing cholesteatoma formation.

## Conclusion

Proper placement of the vascular strip with the skin lining the external auditory canal with approximation of the incision margins is essential to prevent iatrogenic cholesteatoma formation. Close follow-up is essential to prevent any recurrence and diffusion weighted MRI plays a vital role in detection of recurrence.

## References

[1] Cronin SJ, El-Kashlan HK, Telian SA (2014 Sep). Iatrogenic cholesteatoma arising at the bony-cartilaginous junction of the external auditory canal: a late sequela of intact canal wall mastoidectomy. Otol Neurotol.

[2] Dubach P, Mantokoudis G, Caversaccio M (2010 ). Ear canal cholesteatoma: meta-analysis of clinical characteristics with update on classification, staging and treatment. Curr Opin Otolaryngol Head Neck Surg.

[3] Sweeney AD, Hunter JB, Haynes DS, Driscoll CL, Rivas A, Vrabec JT (2017 ). Iatrogenic cholesteatoma arising from the vascular strip. Laryngoscope.

[4] Dubach P, Häusler R (2008 Oct). External auditory canal cholesteatoma: reassessment of and amendments to its categorization, pathogenesis, and treatment in 34 patients. Otol Neurotol.

[5] de Zinis LO, Tonni D, Barezzani MG (2010 ). Single-stage canal wall-down tympanoplasty: long-term results and prognostic factors. Ann Otol Rhinol Laryngol.

[6] Konishi M, Iwai H, Tomoda K (2016 ). Reexamination of Etiology and Surgical Outcome in Patient With Advanced External Auditory Canal Cholesteatoma. Otol Neurotol.

[7] Aikele P, Kittner T, Offergeld C, Kaftan H, Hüttenbrink KB, Laniado M (2003 ). Diffusion-weighted MR imaging of cholesteatoma in pediatric and adult patients who have undergone middle ear surgery. AJR Am J Roentgenol.

[8] Holt JJ (1992 ). Ear canal cholesteatoma. Laryngoscope.

[9] Naim R, Linthicum F Jr, Shen T, Bran G, Hormann K (2005 ). Classification of the external auditory canal cholesteatoma. Laryngoscope.

